# Tuning B cell responsiveness by antigen receptor isotype

**DOI:** 10.18632/oncotarget.4880

**Published:** 2015-07-10

**Authors:** Hassan Jumaa

**Affiliations:** Institute for Immunology, University Hospital Ulm, Ulm, Germany

**Keywords:** Immunology and Microbiology Section, Immune Response, Immunity

The expression of functional B cell antigen receptors (BCRs) is essential for development, survival and activation of B cells. During early developmental stages, immature B cells exclusively express IgM. Cells that pass the immature B cell stage and leave the bone marrow coexpress BCRs of IgM and IgD isotypes, which differ in the usage of the heavy chain (HC) μ versus δ that are generated by alternative poly-adenylation and splicing. It is currently unclear how the prevalent expression of the μHC over δHC at the early stages of B-cell development is reverted in mature B cells, where IgD becomes the dominant antigen receptor. The reason for this tightly regulated expression and the individual function of IgD versus IgM are poorly understood. Another remarkable difference is that μHC can be found both as membrane-associated BCR and soluble antibody, whereas IgD antibodies are barely detectable in the serum and δHC is predominantly part of membrane-associated IgD BCR.

This points to a cell-associated signaling specific function of IgD, which is supported by experiments suggesting that activation IgD as compared with IgM BCR leads to specific kinetics of phosphorylation of intracellular proteins *in vitro* [1]. The role of IgM and IgD was studied *in vivo* by selective expression or deletion of either μHC or δHC in transgenic animal models. This showed that, while IgM and IgD may largely substitute for each other, the development of innate-like B cells was impaired in IgM knockout mice whereas IgD knockout B cells show defects in affinity maturation [2–3].

Continuous exposure to soluble neo-self-antigen in transgenic mice expressing hen-egg-lysozyme (HEL) and the cognate BCR (IgHEL) manifests with downregulation of surface IgM while IgD expression is unaffected [4]. Peripheral B cells from these mice are resistant to activation by soluble HEL and exemplify the original description of B-cell anergy. Remarkably, selective downmodulation or removal of IgM is characteristic for normal mature B cells or human B cells expressing autoreactive receptors [5–6]. While these data suggest a role for IgD in regulating the activation of mature B cells, the underlying molecular mechanism remained unclear.

Using an *in vitro* reconstitution system, model BCRs including the IgHEL were investigated as IgM and IgD receptors bearing the same antigen specificity. Surprisingly, the tested BCRs responded to treatment with low-valence antigens, such as soluble HEL, only when expressed as IgM but not when expressed as IgD BCR. Treatment with multivalent antigens however resulted in comparable activation of all receptors [7].

These data suggested that anergic B cells might not respond to the treatment with soluble monovalent antigens and maintain IgD expression on B cells simply because IgD requires polyvalent antigen for stimulation. Testing this hypothesis on splenic cells revealed that anergic B cells from IgHEL transgenic mice are fully responsive to polyvalent antigen. Characterization of the molecular mechanism in more detail identified the hinge region in the heavy chain of IgD as the essential element for the distinctive IgD function. It seems that the hinge region allows the two arms of IgD to act as pincers that promote binding of low-valence antigen by one IgD, thereby preventing BCR-BCR interaction.

Together, it is tempting to speculate that anergy is a regular step of normal B cell development towards mature B cells and that soluble self-antigens are involved in the generation of mature B cells. Moreover, the increased expression of IgD provides mature B cells with an antigen receptor, which is optimized for activation by multimeric immune complexes and for efficient recruitment into T cell-dependent immune responses. Intriguingly, an additional level of regulation emerges as monovalent antigens may interfere with polyvalent antigens for IgD binding. In fact, soluble HEL prevents the activation of IgHEL splenic cells expressing IgD BCR by multimeric HEL. Thus, it is conceivable that soluble self-antigens, while contributing to the maturation of B cells, block mature B cell activation by interfering with immune complexes containing self-antigen. It seems that the balance between soluble and multimeric antigen in immune complexes is an important parameter for mature B cell activation. This balance might be shifted under conditions of chronic inflammation or infection where immune complexes containing self-antigens may be increased thereby leading to chronic B cells activation and eventually autoimmune diseases or continuous proliferation. This scenario points to the potential use of soluble auto-antigens to control autoimmune diseases or lymphoproliferative disorders if the abnormal cells express IgD. On the other hand, the ratio of soluble versus complex antigen might be a key parameter for the design of protective immunization and vaccination as IgD expression is optimal for recruitment into T cell-dependent immune responses, which include the generation affinity-matured memory cells. Since IgG-type BCRs expressed on memory B cells also contain a hinge region similar to IgD, it is also conceivable that memory B cell responses are also regulated by the ratio of low-valence to multi-valence antigen.

The emerging scenario suggests that the expression of IgD raises the activation threshold, renders cells inducible selectively by complex antigen and directs the cells towards memory responses, while the control by low-valence antigens contributes to B cell maturation and tolerance. On the other hand, the high sensitivity of IgM BCR may be important for stringent selection of early immature B cells and may also confer transformed cells with a receptor isotype that efficiently reacts to multiple stimuli including low-valence antigen.
